# MWCSGA—Multi Weight Chicken Swarm Based Genetic Algorithm for Energy Efficient Clustered Wireless Sensor Network

**DOI:** 10.3390/s21030791

**Published:** 2021-01-25

**Authors:** Nader Ajmi, Abdelhamid Helali, Pascal Lorenz, Ridha Mghaieth

**Affiliations:** 1 Micro-Optoelectronic and Nanostructures Laboratory (LR99ES29), Faculty of Sciences of Monastir, University of Monastir, Environment Street, 5019 Monastir, Tunisia; ajmii.nader@gmail.com (N.A.); abdelhamid.helali@gmail.com (A.H.); ridha.mghaieth123@gmail.com (R.M.); 2 IRIMAS Laboratory/GRTC, University of Haute Alsace, 68008 Colmar, France

**Keywords:** wireless sensor networks (WSNs), clustering, chicken swarm optimization (CSO), genetic algorithm (GA), energy efficient

## Abstract

Nowadays due to smart environment creation there is a rapid growth in wireless sensor network (WSN) technology real time applications. The most critical resource in in WSN is battery power. One of the familiar methods which mainly concentrate in increasing the power factor in WSN is clustering. In this research work, a novel concept for clustering is introduced which is multi weight chicken swarm based genetic algorithm for energy efficient clustering (MWCSGA). It mainly consists of six sections. They are system model, chicken swarm optimization, genetic algorithm, CCSO-GA cluster head selection, multi weight clustering model, inter cluster, and intra cluster communication. In the performance evaluation the proposed model is compared with few earlier methods such as Genetic Algorithm-Based Energy-Efficient Adaptive Clustering Protocol For Wireless Sensor Networks (GA-LEACH), Low energy adaptive Clustering hierarchy approach for WSN (MW-LEACH) and Chicken Swarm Optimization based Genetic Algorithm (CSOGA). During the comparison it is proved that our proposed method performed well in terms of energy efficiency, end to end delay, packet drop, packet delivery ratio and network throughput.

## 1. Introduction

### 1.1. WSN

**Figure 1:**
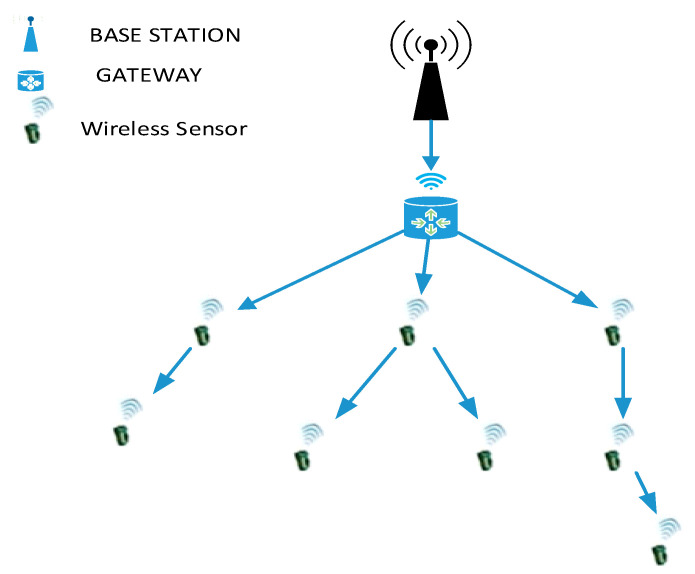
Architectural topologies of wireless sensor networks.

In general, the wireless sensor network (WSN) contains large number of sensor nodes monitored are controlled by the base station (BS). A major process behind the sensor nodes are data transmission, sensing the devices and computation with limited consumption of power factor as well as it is non-rechargeable in nature. Due to these drawbacks there is still a research gap to increase the energy efficiency and to augment network lifetime [1]. WSN sensors undergone real life data processing too much monitoring. Better climate, industrial, etc., monitoring [2]. The general architectural topologies of wireless sensor networks are shown in Figure 1.

**Figure 2:**
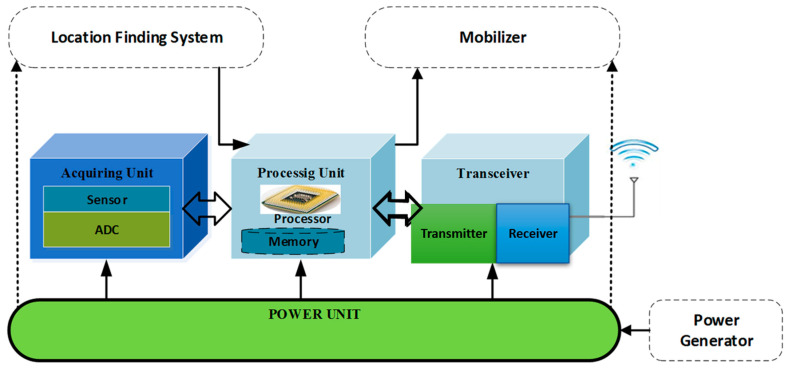
Components of a sensor node.

WSN technology is able to achieve the impossible missions in and around the surface of the earth. As a consequence of enormous success of WSN technology and information communication service, significantly wireless networks transform the globe in to a smaller village in the business world [3]. WSN achieved a new milestone in the following applications such as oceanography, environmental monitoring, agriculture, and engineering. In ad-hoc wireless personal area networks (WPAN) huge numbers of intelligent cost effective battery-powered sensor nodes are produced where it consists of a processor, a radio module, a power supply linked with it [4]. Whereas in WSN, the major component of the sensor nodes is a sensing unit, a processing unit, a transceiver, and a power unit as it is explained in the Figure 2. There are huge architectural models are present in the sensor nodes they are infrared, thermal, seismic, visual which are mainly used for environmental and industrial applications.

- Sensing Unit—This unit is mainly utilized to measure the physical distinctiveness of the surrounding by using the array of sensors.
- Processing Unit—In this section microcontrollers are used to initiate the mission, data processing and control. Because microcontrollers are extremely small in size, they are low powered device and simple, so they are used inside the sensors.
- Transceiver—The process of this unit is to transmit and receive the information in the wireless medium.
- Power source—The process of this unit is to supply the power to all the components in the sensor [5].

Usually, sensor nodes are self-organizing in nature, so it is very suitable for network management. It can create a network by itself, and at the same time guide and monitor the network in an effective way. The major drawbacks are it contains limited power resources, processing ability, and storage space. As so to overcome this communication protocols are used [6].

**Figure 3:**
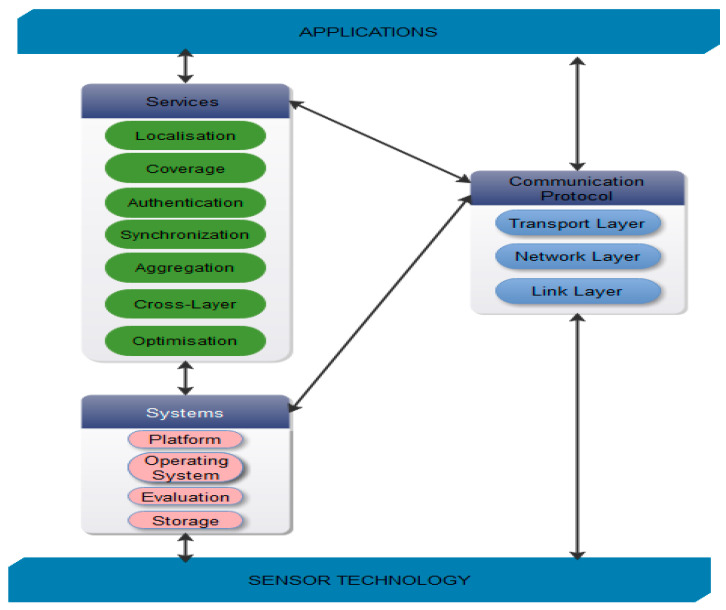
Various issues in a wireless sensor network (WSN)—Broad Classification.

The communication protocols are sub-divided into five sections of layers for the purpose of packet switching as shown in Figure 3. Those are physical, data link, network, transport, and application layers. The primary functionalities of these layers are sensor deployment, management, authentication, aggregation, and storage. The parameters which can be affected due to this process of protocols in protocol stack are energy efficiency, consumption, and latency. In such case optimization becomes essential to reduce the power consumption. New energy efficient protocols with cross layer approaches are introduced to overcome this problem because conventional approaches are not suitable for this process [7,8].

In the other hand, security plays an important role. The major security necessities of wireless sensor network are data confidentiality, data integrity, availability, data freshness, secure localization, time synchronization, and self-organization [9].

### 1.2. Clustering Based Approaches

**Figure 4:**
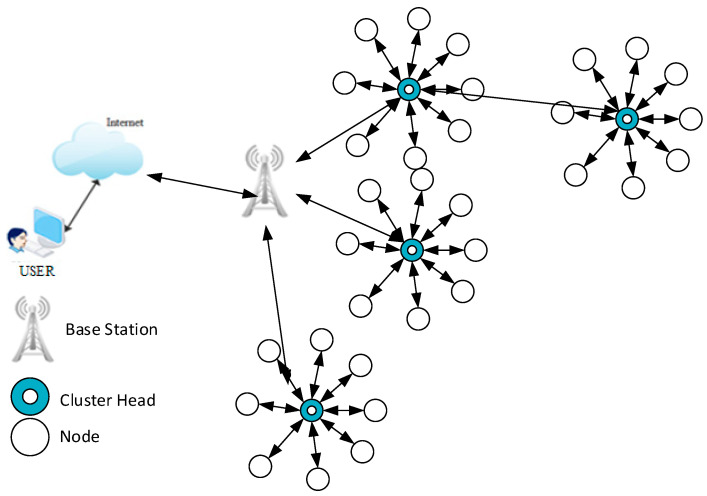
A typical application of a clustered WSN.

Ordinarily, clustering is defined as grouping of sensor nodes and it is referred as cluster. It is a two-layered strategy which separates the network into two levels. For the process of effective communication initially nodes are divided into clusters, and the best node is chosen as a cluster head (CH). This process becomes very essential because a wide diversity of information is furnished in WSN, here clustering is used to connect and aggregate that information which leads to reduce the energy consumption and so to improve the efficiency of the network. A typical application of a clustered WSN is presented in Figure 4.

As Figure 4 shows, layer 1 nodes will do field sensing and data generation then it is transmitted to the CH in the next layer. Here the process of data aggregation is performed finally the information is sent to the BS in a multi hop manner. The major specifications of CH are monitoring the cluster count, cluster size and the cluster communication model such as inter and intra cluster communication. Many earlier research concentrated the CH selection section in order to improve the performance of the network. The key parameters for CH selection are node movement, localization, roles, process of clusters, cluster head selection, algorithm intricacy, and the dynamic nature of the cluster [10,11].

Characteristics of CH:

✓ Existence: The major type of existence of WSN clustering protocols are CH based and non-CH based. Difference of Capabilities: Founded of energy, the homogeneous and heterogeneous networks are created. In the homogeneous network the energy is equally distributed among the nodes and in the heterogeneous network it is randomly distributed [12].
✓ Role: The primary roles are cluster heads are traffic management and data aggregation.
✓ Collision Avoidance: In order to reduce the collision in the network the multi-hop clustering model is introduced. It consists of both the intra-cluster and inter- cluster communication [13].

**Figure 5:**
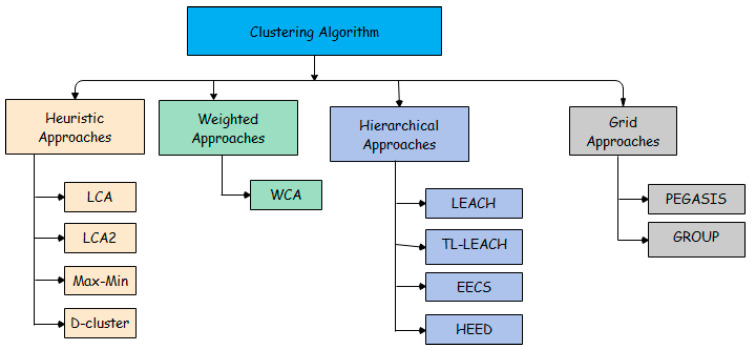
Classification of earlier clustering algorithms.

The Figure 5 demonstrates the classification of earlier clustering algorithms in WSNs. It can clearly be seen that the clustering algorithms is divided into four main approaches which are: Heuristic approaches, Weighted approaches, Hierarchical approaches, and Grid approaches. In the Heuristic approaches, we can cite a few examples of clustering algorithms, such as LCA, LCA2, Max–Min, and D-cluster. On the other hand, the Weighted approaches, here we can talk about, “A Weighted Clustering Algorithm for Mobile Ad Hoc Networks (WCA). While other protocols like PEGASIS and GROUP belong to Grid approaches. On the other hand, in the Hierarchical approaches, this will be the focus of our attention, as it is considered an effective method to reduce energy consumption in the cluster and successfully solve the battery problem. Here, we can introduce different hierarchical routing protocols, such as Low Energy Adaptive Clustering Hierarchy (LEACH), Two-Level Hierarchy for Low Energy Adaptive Clustering Hierarchy (TL-LEACH), Threshold service Energy Efficient sensor Network (TEEN), A Hybrid Protocol for Efficient Routing and Comprehensive Information Retrieval in Wireless Sensor Networks (APTEEN) and Hybrid Energy Efficient Distributed Clustering (HEED).

Furthermore, other protocols include “A Regional Centralized Clustering Routing Algorithm for Wireless Sensor Networks”, “Multiple Sink Network design problem in large scale wireless sensor networks (MSNDP)”, “Energy efficient Dynamic Clustering (EEDC)”, Energy Efficient Clustering using Interconnection (EECI), and Cluster Based Data Aggregation Scheme [14,15,16].

### 1.3. GA Based Approaches

**Figure 6:**
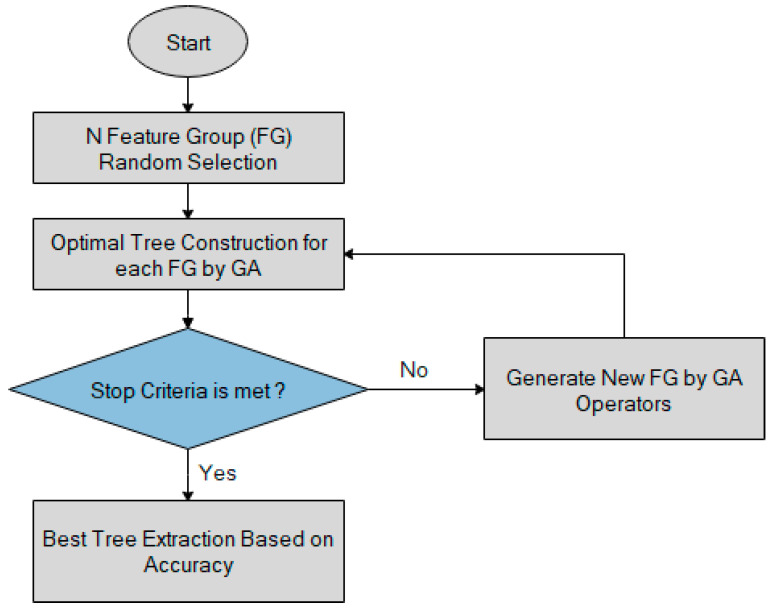
Process of genetic algorithm (GA).

The evolutionary optimization algorithm is classified as a genetic algorithm (GA). Darwinian evolution and survival of the fittest are the core concept of GA. The work flow of GA consists of three steps fitness, survival, and reproduction in an optimized manner [17]. The process of GA is detailed in the Figure 6.

The general operations of genetic optimization algorithm are,

- Step 1—Individuals initial population generation
- Step 2—Fitness value calculation for all the individuals
- Step 3—Repeat
  ○ Fitness based individual’s selection
  ○ Individual’s selection based on genetic operators
  ○ New individual’s fitness calculation
  ○ Update the Current population
- Step 4—End [18]

Several optimization algorithms are developed in earlier days to improve the performance of the network. Some of the genetic algorithm based researches are discussed in this section. In this paper the new hybrid variant of genetic algorithm (GA) is proposed namely adaptive range genetic algorithm (ARGA) which is used to make use of the sample space reduction technique of cohort intelligence (CI) algorithm. As the results of using this model the quality and robustness of the network is improved [19]. In this work, the behavior of genetic algorithms is explained using hyperplastic materials [20]. Genetic algorithm is mainly used for community detection. The author provides genetic encoding methods in this paper which is SGR for community detection as well as the crossover and mutation process are handled effectively [21].

For large-scale optimizations based coevolutionary searches we need additional modifications in genetic algorithm. The author proposed a multi-agent real-coded genetic algorithm (MA−RCGA) for effective frequency exchange process of the network [22]. In the recent days to perform multiple heterogeneous tasks a new model based genetic algorithm is introduced which are a new fuzzy mixed-integer linear programming (FMILP) model and the interval-valued intuitionistic fuzzy entropy weight (IVIFEW) method [23]. For the long-term production planning, production scheduling is a complicated issue. As so to overcome this drawback the maximum flow algorithm (MFA) is combined with genetic algorithm [24]. A stepwise genetic algorithm (SGA) is developed next to improvise the crossover and mutation process in genetic algorithm [25]. In few machine learning based approaches genetic algorithm is used which is to improve the performance of light scattering process [26].

In this paper CPU/FPGA heterogeneous architecture scheduling problem is studied and the author proposed two genetic algorithm based approaches to overcome this issue which are the MPSoC and makespan (minimize the schedule length) [27]. The process of economic optimization is proposed in this paper using genetic algorithm [28,29]. In order to reduce the cost optimization issues hybrid genetic-gravitational search algorithm (HG-GSA) is developed. Hybrid crossover technique is the core idea of the algorithm which is mainly used to reduce the computation cost [30].

## 2. Related Works

### 2.1. GA in WSN

Several optimization algorithms are developed for WSN in earlier days to improve the performance of the network. Some of the genetic algorithm based WSN researches are discussed in this section. For the purpose of solving the link breakage issue in the network a novel genetic algorithm (GA) based on extended sequence and topology encoding (GAEST) is proposed. The major parameters which are considered in this approach are two-tiered multicast protocol, residual energy, mutation-Replace model, crossover model and clustering [31]. In order to reduce energy consumption in the network, a multi-objective energy-aware routing protocol is introduced to achieve the best path selection, namely, multi-objective fractional particle lion algorithm (MOFPL) [32]. In this paper, to increase the lifetime of the network the author developed a hybrid genetic algorithm which is the combination of greedy initialization and bidirectional mutation [33,34].

In case of the energy harvesting-wireless sensor networks (EH-WSN), conventional routing protocols are not effective, because they are not too vital to ensure continuous operation on the network without the need to replace batteries. Hence in this study, a centralized power efficient routing algorithm energy harvesting genetic-based unequal clustering-optimal adaptive performance routing algorithm (EHGUC-OAPR) is proposed. The protocol is mainly used to improve the network lifetime and packet delivery ratio [35]. Other few algorithms are also developed in the same to improve the network lifetime of WSN. They are non-dominated sorting genetic algorithm-II (NSGA-II), a genetic algorithm (GA)-based meta-heuristic model, wireless sensor network dynamic coverage and connectivity problem (WSN-DCCP) and an improved genetic algorithm (ROS_IGA) [36,37,38,39].

### 2.2. GA Based Clustering Approaches

Several genetic optimization algorithms are developed for clustering in earlier days to improve the performance of the network. Some of the genetic algorithm based clustering researches are discussed in this section. In this paper K-clustering algorithm is combined with genetic algorithms to develop a multi-objective genetic algorithm (MOGA) to improve the performance of the network [40]. To improve the network longevity clustering model is constructed in heterogeneous WSN (HWSN) network as well as genetic algorithm-based optimized clustering (GAOC) protocol is introduced with multiple data sinks model called MS-GAOC. It also helps to protect the network from the hop-spot problem [41].

For the effective improvement of accuracy the gravitational emulation local search (GELS) algorithm is combined with the genetic algorithm in this paper [42]. Followed by this, an adaptive clustering-based genetic algorithm (ACGA) is proposed to reduce the total gantry moving distance [43]. For the process of renewable energy sustainability, in this paper fuzzy clustering model is combined with genetic algorithm [44].

### 2.3. GA in Clustering Based WSN

Several genetic optimization algorithms are developed for clustering based WSN in earlier days to improve the performance of the network. Some of the genetic algorithm based clustered WSN researches are discussed in this section. To improve the energy efficiency and to reduce the energy consumption of the network LEACH protocol is combined with genetic algorithm [45,46,47,48,49]. In case of hierarchical routing protocols to improve the lifetime of the WSN network GADA-LEACH protocol is introduced [50]. As so to improve the network availability the author developed a new model, namely, dynamic clustering of heterogeneous WSNs using genetic algorithm (DCHGA) [51]. To perform effective traffic management, load balanced clustering algorithm is combined with genetic algorithm. As the results, the parameters which are improved in the evaluation are energy consumption, energy efficiency, latency, number of CH, rate of convergence, and load balancing [52].

## 3. Fitness Function

In order to improve network lifetime in WSNs. Here, various clustering techniques to achieve this challenge by searching the efficient solution for selection the optimal cluster-head (CHs). The fitness function is the key for all these techniques. The fitness function defines the relative importance of the design. A higher suitability value means a better design. In our study, the fitness function is built that considers the energy consumption and both distance between the cluster head and the BS, and the distance between member nodes and the cluster head. The fitness function F of our method is represented by

(1) $$Fitness\;Function=\overset{\;}{\underset{i}{\sum }}\left(w_{i}*f_{i}\right)\;\forall \;f_{i}\in \left\{E,D\right\}$$

The initial fitness parameters are assigned arbitrary weights ($w_{i}$). In which $w_{1}$ and $w_{2}$ are the weight values of each parameter. These weight values are updated according to the application requirement the total energy consumed to transfer all the collected data to the BS is given by:

(2) $$E=\overset{m}{\underset{i=1}{\sum }}E\left(i,\mathrm{ch}\right)+\overset{n}{\underset{i=1}{\sum }}\left(m*R_{X}\right)+\overset{n}{\underset{i=1}{\sum }}E\left(i,\mathrm{bs}\right)$$

where E(i,ch) is the energy consumption from i th node to the corresponding CH, $\left(m*R_{X}\right)$ represents energy consumed by the CH to receive m message from the member nodes, E(i,bs) represents energy consumption from i th CH to the BS.

The total distances is defined by:

(3) $$D=\overset{m}{\underset{i=1}{\sum }}D\left(i,\mathrm{ch}\right)+\overset{n}{\underset{i=1}{\sum }}D\left(i,\mathrm{bs}\right)$$

D(i,ch) represents distance from node i to the cluster head CH. D(i,bs) is the distance from cluster head i to the BS. “m” and “n” are the cluster member and cluster head, respectively, in first and second term of Equation (2).

## 4. Background on Chicken Swarm Optimization Algorithm

**Figure 7:**
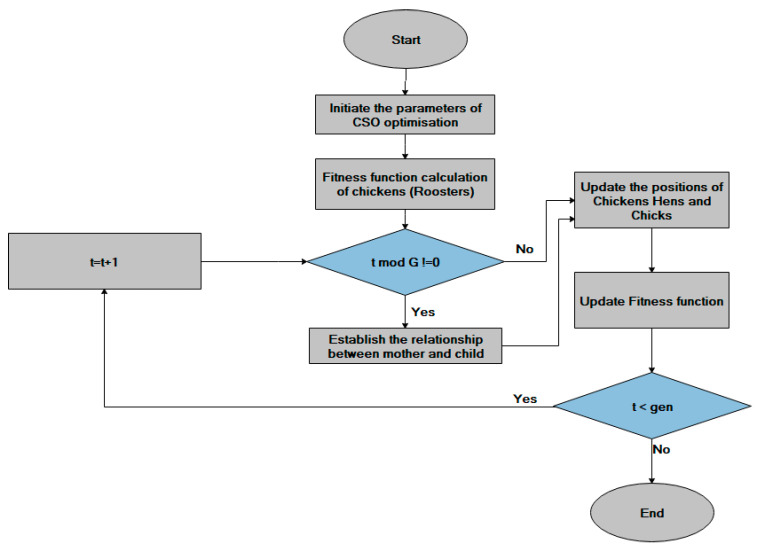
Chicken Swarm optimization Flowchart.

The chicken swarm optimization (CSO) simulated based on the movement of chickens and the performance of chicken swarm. The CSO algorithm consists of various clusters and each cluster contains a leading rooster, hens, and chicks. Each cluster numbers are fixed using the fitness values of the roosters, hens, and chicks. Here the best fitness values are provided to the roosters (chickens). The least fitness values are given to the chicks. The majority in numbers is occupied by the hens and those fitness values are random inside the cluster. The dominant relationship between the mothers to the chicks in the cluster follows the unchanged manner as well as updates the iteration for every instant of time (G). The father to child relation in the cluster among the hens and the child is processed in a random manner. The Figure 7 explains the work flow of CSO optimization algorithm [53].

The movement of chickens mainly depends on the ability to search for food of the different members of the cluster, which is updated according to the fitness values of the initial population. The movement can be formulated as follows:

(1) The movement of the roosters is given by Equation (4)
  (4) $$x_{i,j}^{t+1}=x_{i,j}^{t}\left(1+\mathrm{randn}\left(0,\sigma^{2}\right)\right)$$
  where
  (5) $$\sigma^{2}=\{\begin{array}{cc} 1 & if\;f_{i}\leq f_{k}\\ \exp\left(\frac{f_{k}-f_{i}}{\left|f_{i}+\varepsilon \right|}\right) & Otherwise \end{array}$$
  where $x_{i,j}^{t+1}$ the position of ${\mathrm{rooster}}_{i}$ in $j^{\mathrm{th}}$ dimension during t and t + 1 iteration, $\mathrm{randn}\left(0,\sigma^{2}\right)$ represents a Gaussian random number, the average is 0, and the standard deviation is $\sigma^{2}$. $k\in \left[1,N_{r}\right]$, k≠i and $N_{r}$ is the number of roosters selected, ε is a low-value constant and $f_{i}$ is the fitness value for the corresponding ${\mathrm{rooster}}_{i}$
(2) The movement of the hens is given by Equation (3)
  (6) $$x_{i,j}^{t+1}=x_{i,j}^{t}+S_{1}\times rand\left(x_{r1,j}^{t}-x_{i,j}^{t}\right)+S_{2}\times rand\left(x_{r2,j}^{t}-x_{i,j}^{t}\right)\;\;$$
  where
  (7) $$S_{1}=\exp\left(\frac{f_{i}-f_{r1}}{abs(f_{i})+\varepsilon )}\right)$$
  (8) $$S_{2}=\exp\left(f_{r2}-f_{i}\right)$$
  where r1, r2 $\in \left[1,N_{\;}\right]$, r1≠r2, r1 is the rooster index and r2 is a swarm chicken, which may be a rooster or a hen.
(3) The movement of the chicks is given by Equation (6)
  (3) $$x_{i,j}^{t+1}=x_{i,j}^{t}+FL_{\;}\times \left(x_{m,j}^{t}-x_{i,j}^{t}\right)\;FL\in \left[0,2\right]$$
  where $x_{i,j}^{t+1}$ is the position of the $i^{\mathrm{th}}$ chicks’ mother. FL is a parameter which means the chick will follow its mother. The complete process is explained in Algorithm 1.

**Algorithm 1**-Pseudo code of CSODefine parameters such as population size (popSize), number of generations (gen), the number of roosters (Rn), the number of hens (Hn), the number of chicks (Cn) and the update time steps (G).
Step 1—Initialize the population of chicken as a matrix k;Step 2—Calculates the fitness values for each row in k;Step 3—While (t<gen);Step 4—t = t+1;Step 5—If (t %G == 0)Step 6—Divide k into three groups (rooster, hens, and chicks) according to their fitness valueStep 7—elseStep 8—For i = 1: kStep 9—If (i = rooster); Updates the position of rooster using Eq (4); end ifStep 10—If (i = hen); Updates the position of hen using Eq (6); end ifStep 11—If (i = chick); Updates the position of chick using Eq (9); end ifStep 12—Update the new solution of kStep 13—endStep 14—end

## 5. Background on Genetic Algorithm

The primary process of genetic algorithm is initial population fixing P(k = 0) which is generated in a random manner. The chromosomes are generated by the sequence of genes and those are monitored by definite attributes. Secondly, fitness function is calculated using the values of chromosomes. Then the evolution procedure is carried out where the fit design is generated, and the unfit designs are vanished. This step is continued until the system is completely filled with desirable fitness values. These finally confirmed designs are otherwise called as parents and those are used to produce the designs for future generation called off springs. The evolution process of genetic algorithm is carried out using two sections. They are mutation and crossover. A mutation operator is an arbitrarily process which are generated using the genes of chromosomes and they are selected randomly. In our work, the probability of mutation is $p_{m}\left(k\right)=0.03$. The crossover process takes two specified parent chromosomes to create offspring by swapping operation. We use a single point crossover in which $p_{c}\left(k\right)=0.6$.

The complete process is explained in Algorithm 2 [54].
**Algorithm 2**-Pseudo code of GAStep 1—beginStep 2—initially k = 0;Step 3—Generate Random initial population P(k),Step 4—evaluate initial population P(k) using the Fitness Function;Step 5—while (for unsatisfied conditions) doStep 7—select P(k) from P(k-1);Step 8—k = k + 1;Step 9—Apply single point crossover, $p_{c}\left(k\right)=0.6$.Step 10—Apply mutation with given probability, $p_{m}\left(k\right)=0.03$Step 11—Update the population with new offspringStep 12—endStep 13—Select the best fit chromosome and form the cluster accordingly.

## 6. Proposed Method

This section may be divided by subheadings. It should provide a concise and precise description of the experimental results, their interpretation as well as the experimental conclusions that can be drawn.

### 6.1. System Model

In this research work, the system model consists of a base station (BS) and the sensors (N) which maintains uniform distribution based random deployment in the coverage area. As so to enhance the network connectivity the network is filled with huge number of sensor nodes and are deployed inside the coverage area. The subsections and the hidden details of the network are described as follows.

- The network is totally static which includes the BS and the sensor nodes.
- At the initial stage, all nodes have equal initial energy.
- The BS has no energy limitations its computation energy extremely high.
- According to the coverage area and localization primary cluster head (PCH) and secondary cluster head (SCH) are chosen periodically.
- According to the transmission distance the energy is optimized by the nodes.
- In order to reduce the energy consumption, sleep and wake node concept is initiated in the network.
- Both the cluster heads are multi weighted which it maintains the variable energy level.
- The nodes have the capability to send their address details to its neighbor nodes in the network.
- The BS and PCH are placed within the transmission range in the network.

In general, one hop communication greatly affects the energy. So, p-jump is a better way. Here, the multi weight clustering will be discussed in this paper that uses load balancing in clusters in order to reduce the power consumption and to increase the energy efficiency of WSN.

#### 6.1.1. Energy Model

In our model two types of power loss is used which are free space power loss (d^2^) and multipath fading power loss (d^4^) and according to the transmission distance between the source and the sink the channel model is chosen. In case if this distance is less than its threshold value (d_th_), then it employs the free space model as a channel model or else the multipath fading model is chosen as a channel model. The energy utilization of the data based on the distance factor is mathematically given as follows [55]:

(10) $$E_{TX}\left(l,d\right)=\{\begin{array}{c} l\times E_{energy}+\;l\times E_{tm}\times d^{2},\;if\;d\leq d_{th}\\ l\times E_{energy}+l\times E_{am}\times d^{4},\;if\;d>d_{th} \end{array}$$

where E_energy_ is a total dissipated energy of the circuit per bit, E_tm_ and E_am_ are the transmitter and amplifier model of the network, and d_th_ is the threshold distance of the network and it is given as below in (8)

(11) $$d_{th}=\;\sqrt{\frac{E_{tm}}{E_{am}}}$$

The energy utilization of the sink is given in (9).

E_RX_ (𝑙) = 𝑙 * E_energy_ (12)

#### 6.1.2. Energy Consumption Model

**Figure 8:**
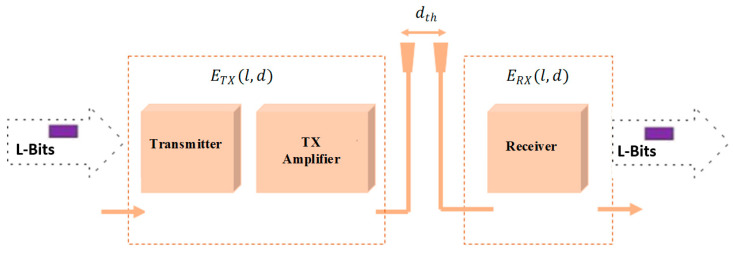
Energy consumption model.

The energy consumption model is explained in the Figure 8 below [56].

The consumed energy of the transmitting node of 𝑙 bits data to cluster head is mathematically expressed in Equation (13).

E_non-CH_ = 𝑙 * E_energy_ + 𝑙 * E_tm_d^2^_cn-CH_ (13)

where d_cn-CH_ = Child node to CH distance

Now the consumed energy of the cluster head CH is expressed in (14).

(14) $$E_{CH}=\left(l\left(\frac{n}{c}-1\right)*E_{energy}+\;\frac{n}{c}*\;E_{con}\right)+\;E_{RX}\left(l,d\right)+\;E_{TX}\left(l,d_{CH-BS}\right)$$

where n is the number of alive nodes in the network, c the number of clusters in the network, E_RX_ is the energy consumption of the cluster head, and d_CH-BS_ is the base station to cluster head distance.

### 6.2. CSO-GA Architecture

Evolutionary algorithms has many classification, genetic algorithm is one among them which is inspired by the natural selection approach. It consist of two main process namely crossover and mutation with present generation included to produce the future generation. In genetic algorithm several individuals are present as so to select the best individual the crossover process is used. Alternatively, to improve the probability of the algorithm to produce more individuals with best fitness function diversity is added to the populations which are done by the process of mutation.

**Figure 9:**
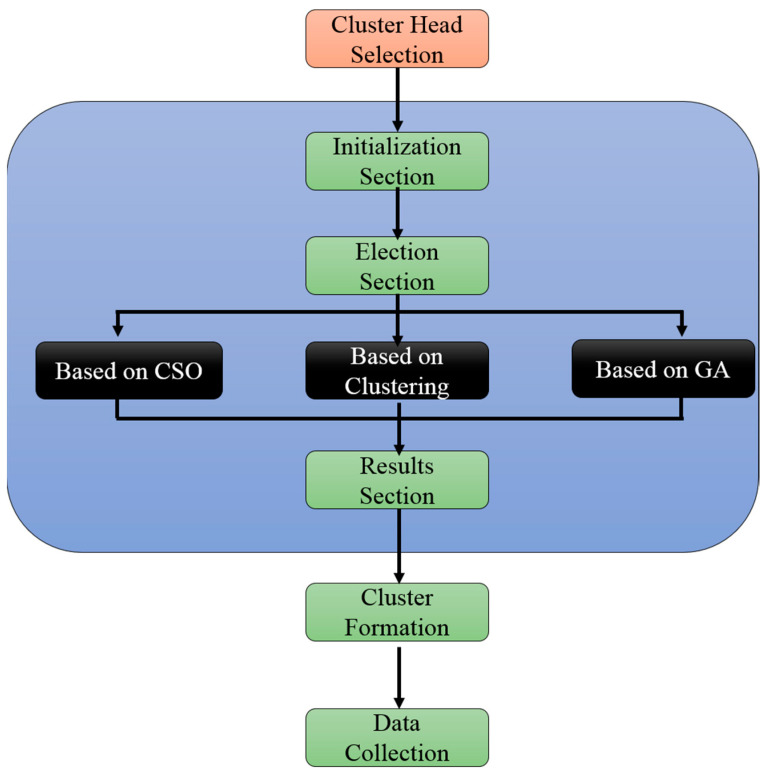
CSO with Genetic Algorithm (CSOGA) architecture.

In our research work, genetic algorithm is used to improve the performance of clustering based chicken swarm optimization algorithm which it includes the major process of genetic algorithm (crossover and mutation). The aim of CSO-GA is to reduce the energy consumption in order to improve the network lifetime. The Figure 9 explains the architecture of CSOGA. The CSOGA algorithm consist of three phases, they are CH selection, cluster formation and data collection.

#### 6.2.1. CSOGA Cluster Head Selection

The cluster head selection is enhanced by using the genetic algorithm crossover and mutation process to maximize the diversity population of the network. This section consists of three processes namely initialization process, election process and ending process. Inputs are initiated in the first section those are CSO parameters, cluster heads count, crossover and mutation. In the ending section best fitness individuals are selected and which greatly helps to the process of CHs selection. In the middle, the election section the roosters are chosen which the individuals are with better fitness function and it is transmitted to the consequent generation. Here the other individuals are hens and chicks; those are undergone to crossover and mutation process. The complete process is explained in Algorithm 3:
**Algorithm 3**-CSOGA**Initialization process:** Step 1: initialize all parameters of CSO, population size, the number of CH (K), the number of (roosters, hens and chicks), crossover $p_{c}\left(k\right)=0.6$, mutation $p_{m}\left(k\right)=0.03$. Further, swarm updating frequency (G) and the maximum number of iteration. **Ending and Selection process:** Step 2: Initialize the population of as a matrix Step 3: Calculates the fitness values for each row in matrix Step 4: While (t<gen); and t = t+1; Step 5: Divide k into three groups (rooster, hens, and chicks) according to their fitness value which are Equation (3), Equation (6), and Equation (9), respectively. Step 6: Compute the fitness value of each row in matrix, and then update the best solution Step 7: else; Apply single point crossover and mutation probability Step 8: Repeat from step 2 to step 7 till reaching the maximum number of iterations **Output process:** Finally, the algorithm converts the output (Best solution) Convert to binary form and return Include the index of the node acting as CH (where, A value indicates that the index of this position is CH)

#### 6.2.2. Cluster Formation

In general, the BS selects the nodes which can be chosen as a cluster heads, it transmit the hello packets to each group to find the cluster head. All the nodes which get the data will transmit reply packets with its address. The node which has the least communication cost and in the perfect localization will be elected as a CH. At last the cluster head is chosen it sends the hello packets to the nodes which are in the coverage area to select the child nodes (CNs).

#### 6.2.3. Data Collection

In this section, the cluster child nodes transmit the data to its CH in the fixed time slot. The data which is transmitted consists of additional information such as node address and initial energy of the node. This information is essential to find the CHs for next round. In order to eliminate the repeated and duplication data the process of data aggregation is used. At the end, the CH transmits the data to its BS alongside with the general information of the child nodes [57].

### 6.3. Multi Weight Clustering Model

The process of multi weight clustering model is given in the algorithm 4. This clustering model works with uniform clustering method as so to reduce the energy consumption and to increase the life time of the network. The minimized energy consumption is achieved by the process of decreasing the distance during the process of communication in WSN. This can be achieved by multi-hoping concept and uniform clustering model. For this, the multi weight clustering follows the centralized model. The major parameters which are concentrated for the multi weight clustering model is localization of nodes and its residual energy. Further, the child node calculation of each cluster head is done. In the simulation section, the analysis is handled among various nodes and location. The multi weight clustering uses load balancing in clusters in order to reduce the power consumption and to increase the energy efficiency of WSN [58].
**Algorithm 4**-Pseudo code of multi weight clustering model:*Begin:* *for every cluster C_s_, repeat*  *for each node n, repeat*        *//hello message sent*    *//Degree Calculation*    $E_{r}=\left\{\left(u,v\right)\;∁\;\frac{V}{D\;\left(u,v\right)}\leq R\right\}$    *Degree = absolute (E)*      *//Energy consumption calculation*        $0.05J<\frac{E_{con}\left(i\right)}{E0}\leq 0.5J$      *//BS distance calculation*        $DistanceBS\left(u\right)=\;\sqrt{{\left(x-\;X_{BS}\right)}^{2}+\;{\left(y-\;Y_{BS}\right)}^{2}}$      *//Weight (Weight)*        $Weight\left(e\right)=\frac{1}{Degree\left(i\right)}+\frac{E_{con}\left(i\right)}{E0}+\frac{DistanceBS\left(i\right)}{DistanceBS\_Max}$    *end*  *if (Weight(i) = min_Weight(i)) then*  *//Cluster Head (CH)*    *St(i) = CH*    *Message (CH approved)*  *else*    *St(i) = ON*    *Message (CH approved)*  *end* *end*

#### Multi Weight Clustering Parameters

In WSN, several parameters are considered for energy consumption, e.g., network mobility, during data transmission, weight of CH node, and network topography. In general one hop communication greatly affects the energy. So, p-jump is a better way in this case. In the upcoming section we will discuss about the weight effect on power consumption. In this model various weight factors are analyzed, and the best weight energy is chosen. The mathematical expression for the node weight in the CH is given as follows.

(15) $$Weight\left(e\right)=\;E_{con}\left(i\right)+\;\frac{1}{D\left(i\right)}+\;n\times Distance\;\left(i\right)$$

The weight of the network can be affected by the values of n, so here we have to test the weight by varying the n values, n = 0, 1, 1/200, which leads to test the performance of the cluster head selection process.

In case 1, if the value of n = 0,

(16) $$Weight\;\left(i\right)=\;E_{con}\left(i\right)+\frac{1}{D\left(i\right)}$$

In case 2, residual energy is also calculated, if the node with highest residual energy is chosen for weight calculation.

In case 2, if the value of n = 1,

(17) $$Weight\;\left(i\right)=\;E_{con}\left(i\right)+\;\frac{1}{D\left(i\right)}+\;Distance\left(i\right)$$

If the node residual energy is given as 0.5 Joules, then the equation given as,

(18) $$0\;\leq \;E_{con}\left(i\right)\leq 0.5\;Joules$$

Here, N is defined as the number of nodes, then the equation becomes,

(19) $$0\;\leq \;\frac{1}{D\left(i\right)}\;\leq \;\frac{1}{N-1}\;\leq 1$$

If the node is approximately placed 10 m away from the base station, then the base station coverage area is 200, so we get,

(20) 10 ≤DistanceBS(i)≤200

Thus, the residual energy and the degree are negligible along with the coverage area of the base station.

In case 3, if the value of n = 1/200, Then the equation becomes,

(21) $$Weight\;\left(i\right)=\;E_{con}\left(i\right)+\;\frac{1}{D\left(i\right)}+\;\frac{DistanceBS}{200}$$

### 6.4. Intra Cluster Communication

In addition to the main cluster head (MCH) a new backup cluster head (BCH) is created in each cluster to effectively utilize the one hop communication process. TDMA and CSMA are used for the data communication in this section. TDMA handles the time slot allocation process among the nodes in the network. As the results all the nodes will transmit the data in the particular instant of time. At the initial condition, the MCH selects its sink node (MCH or BCH) for each and every node inside the cluster. The major step of intra cluster communication is given as follows,

- Sink node determination
- Time slot determination of each node
- Data transmission
- Aggregation

### 6.5. Inter Cluster Communication

In this communication model, all the cluster head will transmit the information to the destination. The process id continued until all the data reaches its destination. In order to transmit the data the concept of CDMA is used. At the initial condition only the MCH and the BCH node is in the wake mode. For inter cluster communication CSMA method is used and to transfer the data inside each cluster the CDMA method is used. The data which is transmitted consists of little additional information such as the iteration count, level information, section information, address, ratio of message that has to be transmitted and the free space details. Each transmission consists of periods which are created using the data size of the node and CH count in the level of the node [59].

## 7. Simulation Environment

The methodology and experimentation for implementing the MWCSGA protocol is discussed. It is extension of CSOGA protocol by doing several modifications and uses network simulation (NS-2) test bed for the performance evaluation of our protocol. Other than CSOGA few earlier methods are also used for performance evaluation which is GA-LEACH and MW-LEACH. Simulation is used to test the protocol several times with various scenarios as well as it is cost effective. Ns2 is basically consists of two languages which is front end of TCL and back end of C++. Network animator (NAM) visualizes the output which is the node deployment of the network in real time. Trace files are used for performance evaluation. The simulation parameters and their values are discussed in the Table 1. The results parameters which are calculated are energy efficiency, energy consumption, end to end delay, packet drop, and throughput and packet delivery ratio.

### 7.1. Energy Efficiency Calculation

**Figure 10:**
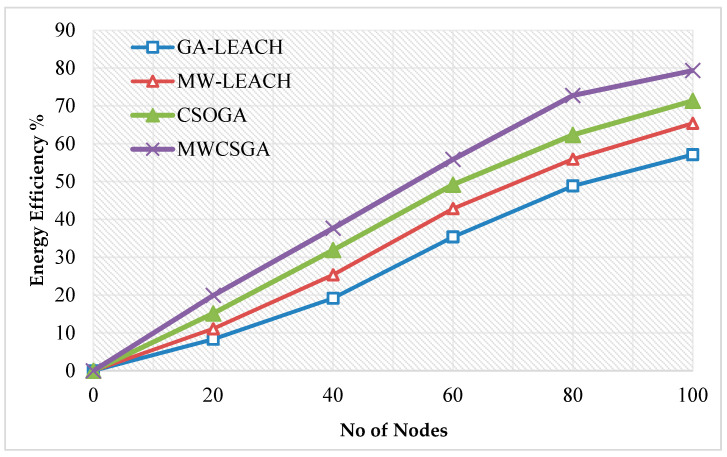
Energy efficiency calculation.

The energy efficiency is the remaining energy at the end of the network data transmission. Figure 10 compares the energy efficiency performance of the nodes for various protocols namely GA-LEACH, MW-LEACH, CSOGA, and MWCSGA. For this scenario, the simulation experiment is carried using 100 nodes. The energy efficiency performance of MWCSGA protocol is 79.32%. Whereas the earlier protocols such as GA-LEACH, MW-LEACH, and CSOGA protocols produces energy efficiency as 57.11%, 65.38%, and 71.34%, respectively. According to analyzes it proves that the performance of MWCSGA protocol performs better when compared with the others.

### 7.2. End to End Delay Calculation

**Figure 11:**
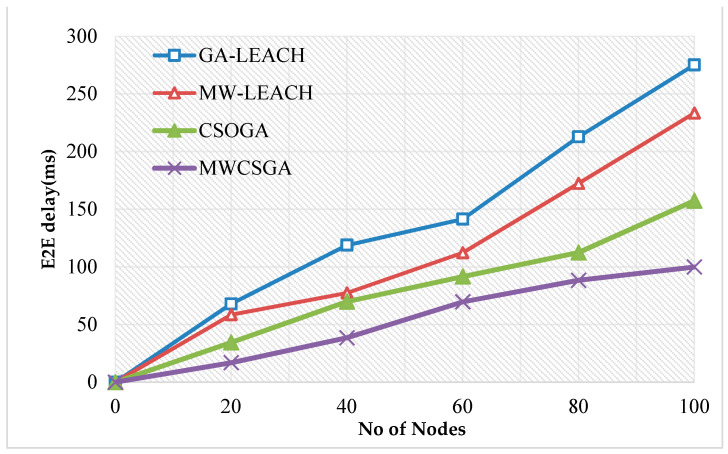
End to end delay calculation.

The End to end delay is the overall delay of the network is the overall delay of the network. Figure 11 compares the end to end delay performance of the nodes for various protocols namely GA-LEACH, MW-LEACH, CSOGA, and MWCSGA. For this scenario, the simulation experiment is carried using 100 nodes. The end to end delay performance of MWCSGA protocol is 99.86 ms. Whereas the earlier protocols such as GA-LEACH, MW-LEACH, and CSOGA protocol produces energy efficiency as 275.12 ms, 233.35 ms, and 157.36 ms, respectively. According to analyzes it proves that the performance of MWCSGA protocol produces low delay when compared with the others.

### 7.3. Packet drop calculation

**Figure 12:**
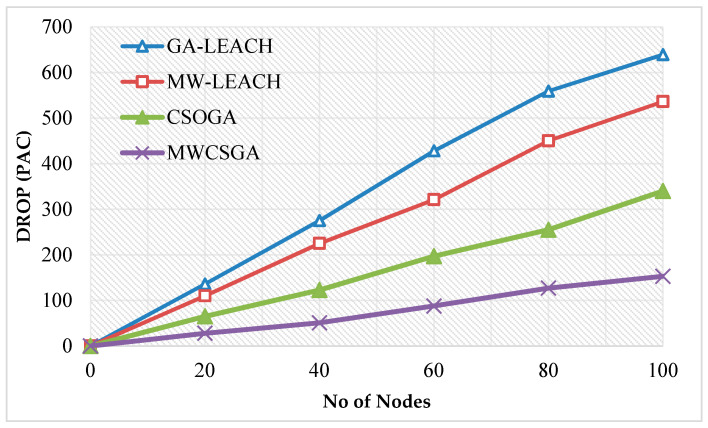
Packet drop calculation.

The packet drop calculation is done using the formula (number packet sent—number of packet received). Figure 12 compares the packet drop performance of the nodes for various protocols namely GA-LEACH, MW-LEACH, CSOGA, and MWCSGA. For this scenario, the simulation experiment is carried using 100 nodes. The packet drop performance of MWCSGA protocol is 153 packets. Whereas the earlier protocols such as GA-LEACH, MW-LEACH, and CSOGA protocol produces energy efficiency as 639, 536, and 340 packets, respectively. According to analyzes it proves that the performance of MWCSGA protocol produces less packet drop when compared with the others.

### 7.4. Network throughput calculation

**Figure 13:**
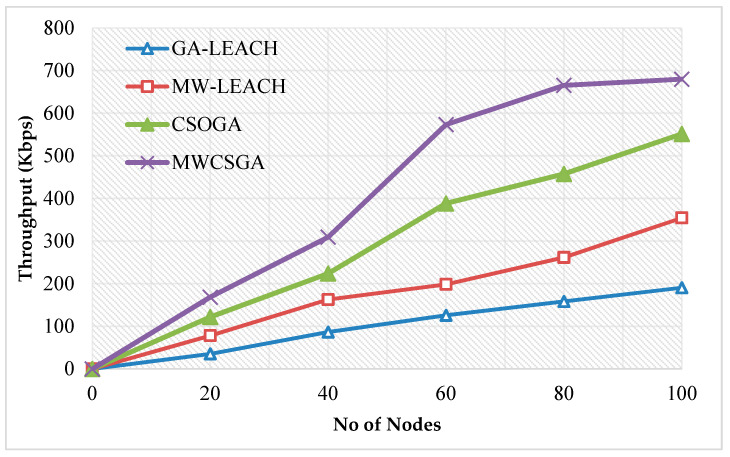
Throughput calculation.

The Network throughput is defined as that the maximum packets received at particular time period. Figure 13 compares the throughput performance of the nodes for various protocols namely GA-LEACH, MW-LEACH, CSOGA, and MWCSGA. For this scenario, the simulation experiment is carried using 100 nodes. The throughput performance of MWCSGA protocol is 679.82 Kbps. Whereas the earlier protocols such as GA-LEACH, MW-LEACH, and CSOGA protocols produce energy efficiency as 190.53 Kbps, 354.37 Kbps, and 551.35 Kbps, respecitvely. According to analyzes it proves that the performance of MWCSGA protocol performs better when compared with the others.

### 7.5. Packet delivery ratio calculation

**Figure 14:**
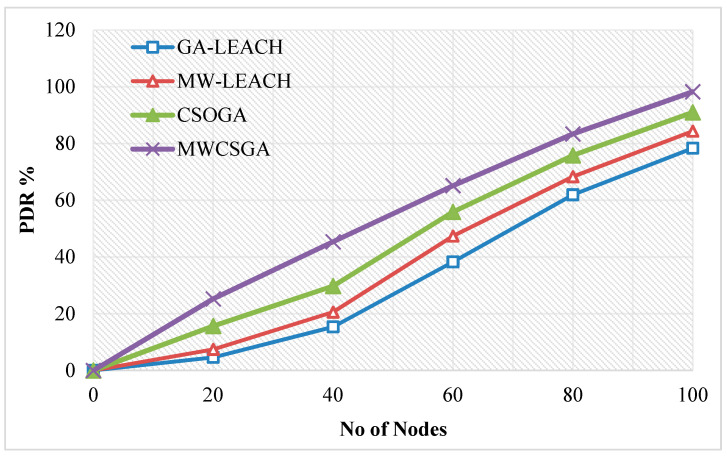
Packet delivery ratio calculation.

The packet delivery ratio is defined as the ratio between numbers of packets received by the destination with respect to number of packet sent by the sender node. Figure 14 compares the packet delivery ratio performance of the nodes for various protocols namely GA-LEACH, MW-LEACH, CSOGA, and MWCSGA. For this scenario, the simulation experiment is carried using 100 nodes. The packet delivery ratio performance of MWCSGA protocol is 98.25%. Whereas the earlier protocols such as GA-LEACH, MW-LEACH, and CSOGA protocols produce energy efficiency as 78.36%, 84.37%, and 90.99%, respectively. According to analyzes it proves that the performance of MWCSGA protocol performs better when compared with the others.

## 8. Conclusions

Hence nowadays WSN is used for several applications there is always an issue occurred due to energy consumption. So as to improve the life span of the network, energy efficiency enhancement is mainly concentrated in our research. Here, the proposed multi weight chicken swarm based genetic algorithm for energy efficient clustering (MWCSGA) protocol helps to increase the energy efficiency during the process of communication in the network. As for the purpose of evaluation our proposed method is compared with few other earlier models namely GA-LEACH, MW-LEACH, and CSOGA. As the results, proposed method performed well in terms of energy efficiency, end to end delay, and throughput and packet delivery ratio. While applying our protocol in large scale based application there is a possibility to receive delay during communication. Then, in order to have formal validation for our results, we planned to apply a normalization model in our future work so that we will be able to compare our algorithm with others recent algorithms in the field such as, P-SEP in [60] and N-SEP [61].

## Figures and Tables

**Table 1:** Simulation parameters details of the proposed model.

Parameters	Values
*Simulator*	NS-2.34
*Simulation Period*	100 ms
*Coverage Area*	1000 × 1000
*No of Nodes*	100
*Initial Energy*	0.5 J
$E_{DA}$	5 nJ/bit/signal
$d_{0}$	87 m
*Packet Size*	4000 bits
E_energy_	50 nJ/bit
*Free space* ($E_{tm})$	10 p J/bit/m^2^
*Multi-path* ($E_{am}$)	0.00013 pJ/bit/m^4^
*Percentage of CHs*	0.05

## Data Availability

Not applicable.

## References

[1] Hassan A.A., Shah W.M., Iskander M.F., Mohamed A.A. (2017). Clustering methods for cluster-based routing protocols in wireless sensor networks: Comparative study. Int. J. Appl. Eng. Res..

[2] Rashid B., Rehmani M.H. (2016). Applications of wireless sensor networks for urban areas: A survey. J. Netw. Comput. Appl..

[3] Worlu C., Jamal A.A., Mahiddin N.A. (2019). Wireless sensor networks, internet of things, and their challenges. Int. J. Innov. Technol. Explor. Eng..

[4] Albaladejo C., Sánchez P., Iborra A., Soto F., López J., Torres R. (2010). Wireless sensor networks for oceanographic monitoring: A systematic review. Sensors.

[5] Tandel H., Shah P.R. (2017). A survey paper on wireless sensor network. Int. J. Sci. Res. Dev..

[6] Yick J., Mukherjee B., Ghosal D. (2008). Wireless sensor network survey. Comput. Netw..

[7] Zhang Y., Wang J., Han D., Wu H., Zhou R. (2017). Fuzzy-logic based distributed energy-efficient clustering algorithm for wireless sensor networks. Sensors.

[8] Akyildiz I.F., Sankarasubramaniam Y., Cayirci E. (2002). Wireless sensor networks: A survey. Comput. Netw..

[9] Sen J. (2009). A survey on wireless sensor network security. Int. J. Commun. Netw. Inf. Secur..

[10] Kaur L., Kad S. (2017). Clustering techniques in wireless sensor network: A review. Int. J. Comput. Appl..

[11] Mitra R., Nandy D. (2012). A survey on clustering techniques for wireless sensor network. Int. J. Res. Comput. Sci..

[12] Rostami A.S., Badkoobe M., Mohanna F., Keshavarz H., Hosseinabadi A.A., Sangaiah A.K. (2018). Survey on clustering in heterogeneous and homogeneous wireless sensor networks. J. Supercomput..

[13] Santhiya S., Thamaraiselvi A. (2013). Survey on energy efficient clustering algorithms for wireless sensor network. Int. J. Latest Trends Eng. Technol. IJLTET.

[14] Devi V.S., Ravi T., Sankaralingam B.P. (2020). Cluster based data aggregation scheme for latency and packet loss reduction in WSN. Comput. Commun..

[15] Kaur S., Mir R.N. (2016). Clustering in wireless sensor networks*—*A survey. Int. J. Comput. Netw. Inf. Secur. IJCNIS.

[16] Malshetty G., Mathapati B. (2019). WSN clustering based on EECI (Energy Efficient Clustering using Interconnection) method. Int. J. Innov. Technol. Explor. Eng. IJITEE.

[17] Arthur J., Bahran R., Hutchinson J., Pozzyi S.A. (2019). Genetic algorithm for nuclear data evaluation applied to subcritical neutron multiplication inference benchmark experiments. Ann. Nucl. Energy.

[18] Adibi M.A. (2019). Single and multiple outputs decision tree classification using bi-level discrete-continues genetic algorithm. Pattern Recognit. Lett..

[19] Iyer V.H., Mahesh S., Malpani R., Sapre M., Kulkarni A.J. (2019). Adaptive range genetic algorithm: A hybrid optimization approach and its application in the design and economic optimization of shell-and-tube heat exchanger. Eng. Appl. Artif. Intell..

[20] Campos J.A., Segade A., Casarejos E., Fernández J.R., Dias J.R. (2019). Hyperelastic characterization oriented to finite element applications using genetic algorithms. Adv. Eng. Softw..

[21] Chen K., Bi W. (2019). A new genetic algorithm for community detection using matrix representation method. Phys. A Stat. Mech. Appl..

[22] Akopov A.S., Beklaryan L.A., Beklaryan A.L. (2019). Parallel multi-agent real-coded genetic algorithm for large-scale black-box single-objective optimisation. Knowl.-Based Syst..

[23] Zhang W., Ding J., Wang W., Zhang S., Xiong Z. (2019). Multi-perspective collaborative scheduling using extended genetic algorithm with interval-valued intuitionistic fuzzy entropy weight method. J. Manuf. Syst..

[24] Paithankar A., Chatterjee S. (2019). Open pit mine production schedule optimization using a hybrid of maximum-flow and genetic algorithms. Appl. Soft Comput. J..

[25] Li J., Zhang H., Luo Y., Deng X., Grieneisen M.L., Yang F., Di B., Zhan Y. (2019). Stepwise genetic algorithm for adaptive management: Application to air quality monitoring network optimization. Atmos. Environ..

[26] Zhaolou C., Fenping C., Xian F., Fenglin X., Chunjie Z., Shixin P. (2019). A hybrid approach using machine learning and genetic algorithm to inverse modeling for single sphere scattering in a Gaussian light sheet. J. Quant. Spectrosc. Radiat. Transf..

[27] Abdallah F., Tanougast C., Kacem I., Diou C., Singer D. (2019). Genetic algorithms for scheduling in a CPU/FPGA architecture with heterogeneous communication delays. Comput. Ind. Eng..

[28] Garcıa J.R., Lopez P.V., Robles D.R., Guaita M. (2019). Cost optimisation of glued laminated timber roof structures using genetic algorithms. Biosyst. Eng..

[29] Chaudhary D., Kumar B. (2019). Cost optimized hybrid genetic-gravitational search algorithm for load scheduling in cloud computing. Appl. Soft Comput. J..

[30] Parinam S., Kumar M., Kumari N., Karar V., Sharma A.L. (2019). An improved optical parameter optimisation approach using Taguchi and genetic algorithm for high transmission optical filter design. Optik.

[31] Zhou J., Cao Q., Li C., Huang R. (2010). A genetic algorithm based on extended sequence and topology encoding for the multicast protocol in two-tiered WSN. Expert Syst. Appl..

[32] Bhardwaj R., Kumar D. (2019). MOFPL: Multi-objective fractional particle lion algorithm for the energy aware routing in the WSN. Pervasive Mob. Comput..

[33] Li J., Luo Z., Xiao J. (2020). A hybrid genetic algorithm with bidirectional mutation for maximizing lifetime of heterogeneous wireless sensor networks. IEEE Access.

[34] Zhang Y., Wu Y.I. (2019). Multiple sources localization by the WSN using the direction-of-arrivals classified by the genetic algorithm. IEEE Access.

[35] Wu Y., Liu W. (2013). Routing protocol based on genetic algorithm for energy harvesting-wireless sensor networks. IET Wirel. Sens. Syst..

[36] El-Sherif M., Fahmy Y., Kamal H. (2018). Lifetime maximization of disjoint wireless sensor networks using multiobjective genetic algorithm. IET Wirel. Sens. Syst..

[37] Manju, Chand S., Kumar B. (2018). Genetic algorithm-based meta-heuristic for target coverage problem. IET Wirel. Sens. Syst..

[38] Martins F.V.C., Carrano E.G., Wanner E.F., Takahashi R.H.C., Mateus G.R., Nakamura F.G. (2014). On a vector space representation in genetic algorithms for sensor scheduling in wireless sensor networks. Evol. Comput..

[39] Yao G., Dong Z., Wen W., Ren Q. (2016). A routing optimization strategy for wireless sensor networks based on improved genetic algorithm. J. Appl. Sci. Eng..

[40] Dutta D., Sil J., Dutta P. (2019). Automatic clustering by multi-objective genetic algorithm with numeric and categorical features. Expert Syst. Appl..

[41] Verma S., Sood N., Sharma A.K. (2019). Genetic algorithm-based optimized cluster head selection for single and multiple data sinks in heterogeneous wireless sensor network. Appl. Soft Comput. J..

[42] Mohammadpour T., Bidgolia A.M., Enayatifar R., SeyyedJavadi H.H. (2019). Efficient clustering in collaborative filtering recommender system: Hybrid method based on genetic algorithm and gravitational emulation local search algorithm. Genomics.

[43] He T., Li D., Yoon S.W. (2018). An adaptive clustering-based genetic algorithm for the dual-gantry pick-andplace machine optimization. Adv. Eng. Inform..

[44] Wang Q., Yang X. (2020). Investigating the sustainability of renewable energy—An empirical analysis of European Union countries using a hybrid of projection pursuit fuzzy clustering model and accelerated genetic algorithm based on real coding. J. Clean. Prod..

[45] Nayak P., Vathasavai B. Genetic algorithm based clustering approach for wireless sensor network to optimize routing techniques. Proceedings of the 7th IEEE International Conference on Cloud Computing.

[46] Bayraklı S., Erdogan S.Z. (2012). Genetic algorithm based energy efficient clusters (GABEEC) in wireless sensor networks. Procedia Comput. Sci..

[47] Hussain S., Matin A.W., Islam O. (2007). Genetic algorithm for hierarchical wireless sensor networks. J. Netw..

[48] Khalil E.A., Attea N.A. (2011). Energy-aware evolutionary routing protocol for dynamic clustering of wireless sensor networks. Swarm Evol. Comput..

[49] Liu J.L., Ravishankar C.V. (2011). LEACH-GA: Genetic algorithm-based energy-efficient adaptive clustering protocol for wireless sensor networks. Int. J. Mach. Learn. Comput..

[50] Bhatia T., Kansal S., Goel S., Verma A.K. (2016). A genetic algorithm based distance-aware routing protocol for wireless sensor networks. Comput. Electr. Eng..

[51] Elhoseny M., Elleithy K., Elminir H., Yuan X., Riad A. (2015). dynamic clustering of heterogeneous wireless sensor networks using a genetic algorithm, towards balancing energy exhaustion. Int. J. Sci. Eng. Res..

[52] Kuila P., Gupta S.K., Jana P.K. (2013). A novel evolutionary approach for load balanced clustering problem for wireless sensor networks. Swarm Evol. Comput..

[53] Walid Osamy W., El-Sawy A.A., Salim A. (2020). CSOCA: Chicken swarm optimization based clustering algorithm for wireless sensor networks. IEEE Access.

[54] Kulandaivel R., Periyanayagi S., Susikala S. (2012). Performance comparison of WSN &WSAN using genetic algorithm. Procedia Eng..

[55] Daneshvar S.M., Mohajer P.A., Mazinani S.M. (2017). Energy-efficient routing in wsn: A centralized cluster-based approach via grey wolf optimizer. IEEE Access.

[56] Zhao Z., Shi D., Hui G., Zhang X. (2019). An energy-optimization clustering routing protocol based on dynamic hierarchical clustering in 3D WSNs. IEEE Access.

[57] El Alami H., Najid A. (2019). ECH: An enhanced clustering hierarchy approach to maximize lifetime of wireless sensor networks. IEEE Access.

[58] El Khediri S., Khan R.U., Nasri N., Kachouri A. (2020). MW-LEACH: Low energy adaptive clustering hierarchy approach for WSN. IET Wirel. Sens. Syst..

[59] Farsi M., Mahmoud Badawy M., Moustafa M., Ali H.A., Abdulazeem Y. (2019). A congestion-aware clustering and routing (CCR) protocol for mitigating congestion in WSN. IEEE Access.

[60] Vinueza Naranjo P.G., Shojafar M., Mostafaei H., Pooranian Z., Baccarelli E. (2017). P-SEP: A prolong stable election routing algorithm or energy-limited heterogeneous fog-supported wireless sensor networks. J. Supercomput..

[61] Vinueza Naranjo P.G., Shojafar M., Mostafaei H., Pooranian Z., Baccarelli E. A new stable election-based routing algorithm to preserve aliveness and energy in fog-supported wireless sensor networks. Proceedings of the IEEE International Conference on Systems and Cybernetics.

